# The Anti-Metastatic Potential of Aronia Leaf Extracts on Colon Cancer Cells

**DOI:** 10.3390/nu16234110

**Published:** 2024-11-28

**Authors:** Katarzyna Owczarek, Miłosz Caban, Dorota Sosnowska, Dominika Kajszczak, Urszula Lewandowska

**Affiliations:** 1 Department of Biochemistry, Faculty of Medicine, Medical University of Lodz, 92-215 Lodz, Poland; milosz.caban@stud.umed.lodz.pl (M.C.); urszula.lewandowska@umed.lodz.pl (U.L.); 2 Institute of Molecular and Industrial Biotechnology, Faculty of Biotechnology and Food Sciences, Lodz University of Technology, 90-530 Lodz, Poland; dorota.sosnowska@p.lodz.pl (D.S.); dominika.kajszczak@p.lodz.pl (D.K.)

**Keywords:** aronia leaf extract, *Aronia melanocarpa*, purification, polyphenolics, colorectal cancer, metalloproteinase-2, metalloproteinase-9, invasion, migration

## Abstract

Background/Objectives: Numerous studies have demonstrated the health benefits of polyphenols found in aronia fruits; however, little is known about how aronia leaf polyphenols impact colorectal cancer (CRC). This study aimed to evaluate the in vitro anti-metastatic and anti-invasive activity of crude aronia leaf extract (ACE) and purified phenolic-rich aronia leaf extract (APE) against two CRC cell lines (SW-480 and HT-29). Methods: Migration and invasion potential of ACE and APE were evaluated. Moreover, ELISA and gelatin zymography were performed to detect translational and activity changes in CRC cells after aronia extracts treatment. Results: We found that a 100 µg/mL concentration of ACE and APE almost entirely downregulated the migration and invasion of SW-480 cells, showing greater effectiveness than HT-29 cells. The observed inhibition was concentration-dependent and statistically significant. Additionally, extracts reduced the product of MMP-2 and MMP-9 gene expression at the protein level and simultaneously inhibited the activity of both MMPs. An APE at 300 µg/mL for SW-480 and 600 µg/mL for HT-29 resulted in a notable reduction in MMP-2 protein synthesis by 72% and 50%, respectively. In contrast, MMP-9 protein synthesis decreased by 48% and 59% in HT-29 cells treated with 300 µg/mL and 600 µg/mL of ACE, respectively. The levels of gelatinase activity were similar for both CRC lines, and the APE tested at a concentration of 300 µg/mL reached almost the IC_50_ value after 48 h of incubation. Conclusions: Based on the presented results, we provided an experimental foundation for future in vitro and in vivo studies on the potential effects and activities of aronia leaves.

## 1. Introduction

The incidence of colon cancer (CRC) is on the rise globally, primarily due to lifestyle and environmental factors, particularly in highly developed countries [1]. From a public health perspective, CRC is the leading cause of cancer-related deaths and the second most common gastrointestinal malignancy [2,3,4]. It has been estimated that over half of CRC patients develop metastasis, with the majority of tumors spreading to the liver (80% to 90%) [5]. About 20% of newly diagnosed CRC patients already have metastatic disease, and an additional 25% of patients with localized disease will develop metastases in the future [6].

The complex mechanisms by which CRC cells form metastases require further investigation. Exactly how cancer cells acquire and maintain metastatic properties remains unclear. It is essential to identify the factors that regulate metastasis, including intracellular elements such as genetic abnormalities, tumor cell heterogeneity, and epithelial-mesenchymal transition (EMT) and aspects of the tumor microenvironment. Current treatments for CRC often lead to undesirable side effects, and there is a notable increase in resistance to systemic chemotherapy [7]. Therefore, there is an urgent need for new therapies to be tested in animal models and clinical trials to improve patient outcomes.

It is well established that cancer cells often spread from the primary tumor to distant sites early in the disease, long before the tumor is diagnosed [5]. These events are observed at the beginning of metastasis and associated with developing a more motile phenotype typical of mesenchymal cells, marked by enhanced migratory ability, increased invasiveness, and the destabilization of the production of extracellular matrix (ECM) components [7,8]. The breakdown of the ECM is a critical part of how cancer cells invade and migrate, and this process relies on the action of numerous enzymes. Matrix metalloproteinases (MMPs) are a significant class of enzymes involved in the degradation of collagen and proteins in the ECM [9]. The changing nature of the tumor microenvironment during cancer progression influences both the availability of substrate resources and the expression of MMPs.

The biological action and regulation of MMPs’ molecular mechanisms in various critical aspects of CRC progression remain insufficiently understood. Nevertheless, research has indicated that the mRNA, protein, and/or activity levels of two specific MMPs, MMP-2 and MMP-9, are significantly upregulated in CRC tissues (even 10-fold within the invasive regions of colon cancer specimens) compared to normal colonic mucosa [10,11]. Most MMPs are secreted as inactive zymogens and become modulated in the extracellular space in response to cytokine stimulation and growth factors. Key stimulators of MMP include tumor necrosis factor-alpha (TNF-α), interleukins (IL), epidermal growth factor (EGF), vascular endothelial growth factor (VEGF), and basic fibroblast growth factor (bFGF) [12,13,14,15,16]. This deregulation involves the activation of zymogen precursors, interactions with specific ECM components, internalization processes, and inhibition by tissue inhibitors of metalloproteinases (TIMPs). Both gelatinases (MMP-2 and MMP-9) are closely associated with cancer invasion and progression, and their elevated expression has been connected to lower survival rates among colon cancer patients [17,18]. MMP-9 plays a crucial role in inducing EMT by proteolytically affecting E-cadherin, leading to the simultaneous loss of other epithelial markers like vimentin or Snail. Conversely, the downregulation of E-cadherin results in the upregulation of MMP-2, which is also expressed in relation to HIF-1α, a factor involved in the hypoxic response [19,20]. 

Therefore, additional research and new therapeutic solutions are necessary to better understand the molecular mechanisms of MMP pathobiology in preventing and treating CRC.

Numerous studies have suggested that plant polyphenols may significantly prevent CRC through epigenetic mechanisms [21]. Based on epidemiological, preclinical, and clinical evidence, these compounds have demonstrated toxic effects on CRC cells and can increase sensitivity to chemo- and radiotherapies.

Furthermore, most cytotoxic drugs affect both cancerous and healthy cells, causing side effects such as bone marrow suppression, hair loss, drug resistance, gastrointestinal lesions, neurologic dysfunction, and cardiac toxicity [22]. Therefore, polyphenolic compounds offer many advantages over synthetic drugs, owing to their safety, nutritional benefits, low cost, and less toxicity [23].

Previous reports have indicated the anti-metastatic potential of different leaf extracts from *Ginko biloba*, *Tithonia diversifolia*, *Coriandrum sativum*, *Ocimum sanctum*, or *Crataegus aronia* in many types of cancer [24,25,26,27,28].

This was one of the reasons why chokeberry leaf extract was chosen for testing.

Available research has well documented the health-promoting properties of aronia, correlating with the high content of polyphenols in fruits. The key benefits of chokeberry fruits include their antioxidant, anti-inflammatory, anti-diabetic, and hepatoprotective properties. Additionally, it supports regulating cholesterol, lowers blood pressure, prevents platelet aggregation, enhances immune system function, and offers protection to the skin against UV radiation [29,30]. Although chokeberry by-products, such as leaves or pomace, have not been extensively studied, they may be equally valuable sources of these bioactive substances. Studies have shown that chokeberry leaves contain the highest levels of polyphenols compared to the fruit and pomace of the plant (twice as high as in the fruit and approximately three times higher than the pomace) [31,32]. The aronia leaves exhibit the most significant antioxidant potential among these three materials, attributed to their considerable polyphenol content [29,31,32]. The composition of polyphenols is similar across the fruit, leaves, and pomace. However, chlorogenic acid and quercetin derivatives (especially rutin and isorhamnetin) are predominantly found in the leaves, while anthocyanins are abundant in the fruit [29,31,32,33]. This highlights the importance of utilizing these by-products and their potential applications in food, nutraceuticals, and pharmaceuticals. Despite containing substantial quantities of polyphenols, they are often discarded as waste.

Our previous study demonstrated the antioxidant and cytotoxic effects of aronia leaf crude phenolic extract (ACE) and purified phenolic-rich extract (APE) on human colon cancer cells (SW-480 and HT-29) without significantly affecting the growth of normal intestinal cells (CCD 841 CoN) [33]. Other research confirmed that chokeberry leaves have antioxidant, antiradical, hypoglycemic, anti-neurodegenerative, antibacterial, and cytotoxic properties [21,29,31,34,35,36].

Based on this evidence, our study aimed to investigate the in vitro potential of aronia leaf extracts in preventing the migration and invasion of colon cancer for the first time. We also evaluated the protein synthesis and gelatinolytic activity level to determine if the observed results were linked to the inhibition of matrix metalloproteinases (MMP-2 and MMP-9).

## 2. Materials and Methods

### 2.1. Chemicals and Research Materials

All the reagents, cell culture medium, supplements, and (-)-epigallocatechin-3-gallate (EGCG) were purchased from Sigma-Aldrich (St. Louis, MO, USA). Ultrapure water (Simplicity^TM^ Water Purification System, Millipore, Marlborough, MA, USA) was used to prepare all solutions. The research material consisted of aronia leaf crude phenolic extract (ACE) and purified phenolic-rich extract (APE), which were used in a previous study [33]. The total phenolic content (TPC) of each extract was as follows: 28.93 mg/g (ACE) and 360.59 mg/g (APE) [33]. To sum up, the compositions of the extracts are presented in Table 1.

### 2.2. Cell Cultures and Treatments

The human epithelial colorectal adenocarcinoma cell lines (SW-480 and HT-29) used in this study were obtained from the American Type Culture Collection (ATCC; ref: CCL-228, HTB-38, CRL-1790) (LGC Standards, Kiełpin, Poland). The cells were grown in RPMI-1640 medium (SW-480) and DMEM (HT-29) with the addition of 10% FBS, 2 mM L-glutamine, 50 U/mL penicillin, and a 50 µg/mL streptomycin mixture. The cells were incubated at 37 °C in a humidified atmosphere with 5% CO_2_. Based on each experimental design, the cells were placed in a growth medium containing 10% FBS for 24 h at an appropriate density. Next, the cell culture medium was removed and replaced with a 3% FBS medium with or without tested extracts. An EGCG concentration of 50 µM for SW-480 or 100 µM for HT-29 was used as a positive control, as a natural green tea compound with proven anticancer and anti-metastatic activity [37,38]. In addition, the MMP-9 protein level was determined in HT-29 cells by stimulating them with 50 ng/mL tetradecanoylphorbol acetate (TPA) and 50 ng/mL TNF-α. Concentrations of the stimulants were chosen experimentally.

### 2.3. Transwell Migration and Invasion Assay

The migration or invasion of SW-480 and HT-29 cells was evaluated using a Transwell assay. Briefly, 1.0 × 10^5^ SW-480 or 1.5 × 10^5^ HT-29 cells in serum-free medium were added into the top chambers and incubated with or without aronia leaf extracts (50 and 100 μg/mL) or EGCG (50 µM for SW-480 or 100 µM for HT-29) as the positive control. Additionally, HT-29 cells were stimulated with 50 ng/mL TNF-α/TPA. The chambers coated with Matrigel (6.5 mm insert; 8.0 µm polycarbonate membrane; Corning Incorporated, Corning, NY, USA) were used for the invasion assays. Subsequently, the lower chambers were filled with a complete medium (containing 10% FBS) to act as a chemoattractant. After 48 h (SW-480) or 96 h (HT-29) incubation at 37 °C in a 5% CO_2_ incubator, migratory/invasive cells that passed through the membranes were stained with crystal violet solution for 10 min, photographed, and measured using NIH ImageJ analysis software version 1.53 a). To remove non-migrated cells, the upper surface of the membrane was gently scraped with a cotton swab. The experiments were repeated in triplicate wells.

### 2.4. ELISA

ELISA assessed the expression of the MMP-2 and MMP-9 proteins. HT-29 and SW-480 cells were seeded in 96-well plates (1.0 × 10^4^ cells/well) in a growth medium with 10% FBS for 24 h. Next, after medium removal, cells were suspended in a medium with 3% FBS with or without aronia leaf extracts (200 and 300 μg/mL for SW-480 and 300 and 600 μg/mL for HT-29) for 48 h. Positive control samples were treated with EGCG (50 µM for SW-480 and 100 µM for HT-29). To determine MMP-9 protein expression in HT-29, cells were stimulated with a combination of 50 ng/mL TNF-α/TPA. The protein level of MMP-2 was determined by using an ELISA Kit for matrix metalloproteinase 2 (Clone Cloud Corp., Katy, TX, USA). Additionally, MMP-9 levels were examined with the RayBio^®^ Human MMP-9 ELISA Kit (RayBiotech Life, Inc., Peachtree Corners, GA, USA) according to the manufacturer’s protocol.

### 2.5. Gelatin Zymography and Quantitative Analysis of MMP-2 and MMP-9 Secretion

SW-480 or HT-29 cells (1.0 × 10^4^) were harvested, suspended in the growth medium mentioned above, and seeded on 96-well plates. After 24 h, the cells were washed twice with PBS and then suspended in a medium containing 3% FBS. Various concentrations of aronia leaf extracts (200, 300, and 500 µg/mL for SW-480 and 300, 600, and 900 µg/mL for HT-29 cells) and EGCG (50 µM or 100 µM) were added for the next 24 and 48 h. Additionally, to assess the effect of aronia extracts on MMP-9 activity in HT-29 cells, MMP-9 expression was induced with 50 ng/mL TNF-α/TPA. After harvesting, conditioned media were stored at –20 °C for further analysis. Secretion of MMP-2 and MMP-9 was evaluated in 10% SDS- polyacrylamide gel containing 1 mg/mL gelatin under non-reducing conditions. After electrophoresis, the gels were washed in a 2.5% Triton X-100 solution for one hour and then incubated overnight at 37 °C in calcium assay buffer (1% Triton X-100, 50 mM Tris–HCl pH 7.4, and 5 mM CaCl_2_). Next, the gels were stained for 1 h in a solution containing 0.1% amido black, 7% acetic acid, and 20% ethanol. Each metalloproteinase was visualized as transparent bands against the dark blue background of the amido black-stained gels. Zymographic bands were quantified using GelDoc ™EQ system with Quantity One software version 4.4.1 (Bio-Rad Laboratories, Inc., Hercules, CA, USA).

### 2.6. Statistical Analysis

The analysis of variance (one-way ANOVA) was performed using PRISM 9.0 (GraphPad Software Inc., La Jolla, CA, USA). The data are presented as mean ± SEM in the figure legends, with the number of independent experiments indicated. The Newman–Keuls post hoc test was used for multiple comparisons to identify statistically significant differences between variables (*p* < 0.05).

## 3. Results

### 3.1. Aronia Leaf Extract Composition

The phenolic composition of aronia leaf extracts (ACE and APE) examined in the present work was described in detail earlier by Owczarek et al. [33]. Briefly, 25 and 42 phenolic compounds were detected by the UPLC technique in the ACE and APE, respectively, including phenolic acids, flavonols, and anthocyanin pigment. The predominant compounds in the ACE and APE, respectively, were chlorogenic acid (49.59% and 35.86%) and quercetin-3-rutinoside (6.07% and 9.12%). A detailed summary of the compositions of the extracts is presented in Table 1.

### 3.2. Effect of Aronia Leaf Extracts on Colon Cancer Cells Migration

**Figure 1:**
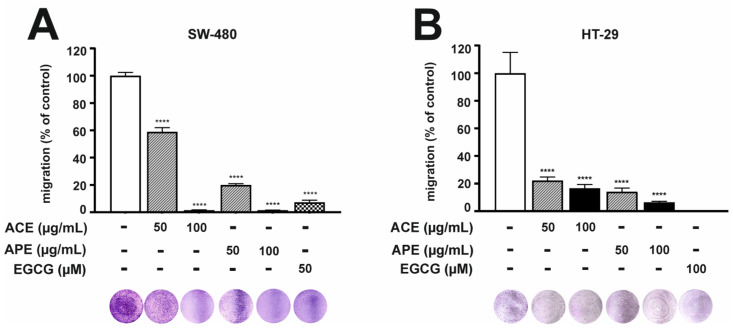
Aronia leaf extracts modulated the migration of human SW-480 (**A**) and HT-29 (**B**) cells. The inhibitory effects of ACE and APE (50 and 100 µg/mL) on the migration of CRC cells were determined using a Transwell assay. Cells were seeded in inserts with a chemoattractant in the lower chamber. After 48 h (SW-480) or 96 h (HT-29) incubation, migratory cells that passed through the membranes were stained, photographed, and measured using NIH ImageJ analysis software. Negative and positive controls were obtained from untreated SW-480 cells (CTRL) and cells treated with 50 µM EGCG. At the same time, negative and positive controls for HT-29 were obtained after stimulation with 50 ng/mL TNF/TPA (CTRL) and with 100 µM EGCG, respectively. Each value represents mean ± SEM, n = 3 independent experiments (each performed in duplicate). Significance of differences between means: **** *p* < 0.0001 versus CTRL (one-way ANOVA followed by Newman–Keuls post hoc test).

The anti-migration effect of aronia leaf extracts in CRC cells was detected by using a Transwell assay. Cells were exposed to 50 or 100 µg/mL of aronia leaf extracts for 48 h (SW-480) or 96 h (HT-29). A higher concentration of tested extracts totally inhibited CRC cells’ migration potential. As depicted in Figure 1, ACE and APE at 50 μg/mL significantly decreased cellular migration by 41% and 80% in SW-480 cells (Figure 1A) and by 77% and 86% in HT-29 cells (Figure 1B), respectively, compared to the migration rate in untreated cells. In addition, the effect was concentration-dependent. The aronia extracts inhibited the migration of cancer cells even more effectively at a concentration of 100 µg/mL.

### 3.3. Effect of Aronia Leaf Extracts on Colon Cancer Cells Invasion

**Figure 2:**
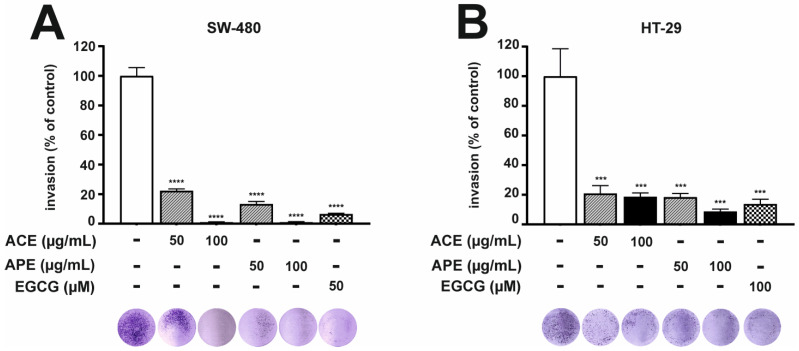
Aronia leaf extracts modulated the invasion of human SW-480 (**A**) and HT-29 (**B**) cells. The inhibitory effects of ACE and APE (50 and 100 µg/mL ) on the invasion of CRC cells were determined using a Transwell assay. Cells were seeded in Matrigel-coated inserts with a chemoattractant in the lower chamber. After 48 h (SW-480) or 96 h (HT-29) incubation, invaded cells that passed through the membranes were stained, photographed, and measured using NIH ImageJ analysis software. Negative and positive controls were obtained from untreated SW-480 cells (CTRL) and cells treated with 50 µM EGCG. In contrast, negative and positive controls for HT-29 were obtained after stimulation with 50 ng/mL TNF/TPA (CTRL) and 100 µM EGCG, respectively. Each value represents mean ± SEM, n = 3 independent experiments (each performed in duplicate). Significance of differences between means: *** *p* < 0.001, **** *p* < 0.0001 versus CTRL (one-way ANOVA followed by Newman–Keuls post hoc test).

The aronia leaf extract treatments suppressed the ability of CRC cells to invade through a Matrigel basement membrane. Cells were exposed to 50 and 100 µg/mL of aronia leaf extracts for 48 h (SW-480) or 96 h (HT-29). As in the case of migration, the obtained effect was concentration-dependent. Both aronia extracts at a concentration of 50 μg/mL inhibited the invasion potential of SW-480 cells by 77% and 86% (Figure 2A) and HT-29 cells by 79% and 82% (Figure 2B) after ACE and APE treatment, respectively. Notably, in the case of HT-29 cells, these results were similar to those of EGCG, a polyphenolic compound known for its anticancer properties. As in the case of migration, the concentration of both extracts (100 μg/mL) that was twice as high almost completely inhibited the invasive ability of SW-480 cells (Figure 2A). We did not present results for higher concentrations of the extracts (>100 µg/mL) because both cell lines’ migratory and invasive potential was completely inhibited.

### 3.4. Effect of Aronia Leaf Extracts on MMP-2 and MMP-9 Synthesis—ELISA

**Figure 3:**
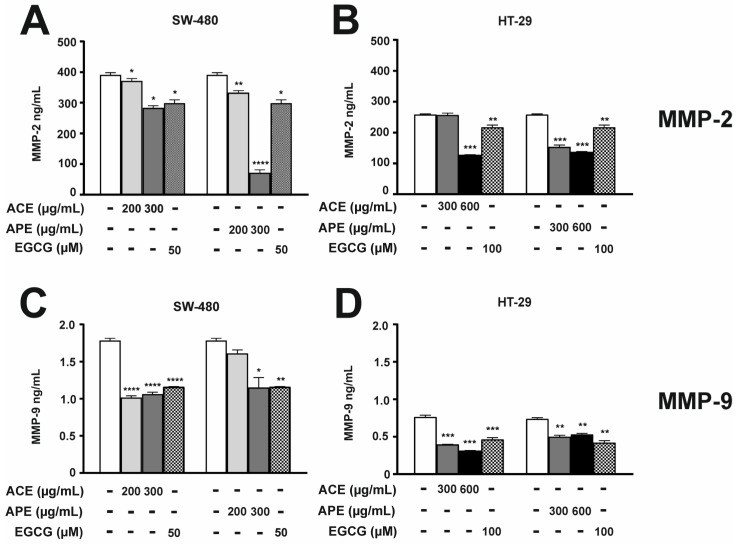
Aronia leaf extracts modulated the product of MMP-2 and MMP-9 gene expression at the protein level, which was determined using ELISAs. After 48 h of incubation, the level of both metalloproteinases in SW-480 cells (**A**,**C**) was examined after chokeberry extracts treatment at concentrations of 200 and 300 µg/mL, whereas in HT-29 cells (**B**,**D**) at concentrations of 300 and 600 µg/mL. Negative controls for SW-480 cells (CTRL) were obtained from untreated cells. In the case of HT-29 cells, negative controls were obtained from cells stimulated with 50 ng/mL TNF/TPA (CTRL). Both cell lines were cultured in a 3% FBS medium. An amount of 50 or 100 µM EGCG was used as a positive control for SW-480 or HT-29 cells. Each value represents mean ± SEM, n = 3 independent experiments (each performed in duplicate). Significance of differences between means: * *p <* 0.1, ** *p* < 0.01, *** *p* < 0.001, **** *p* < 0.0001 versus CTRL (one-way ANOVA followed by Newman–Keuls post hoc test).

Chokeberry leaf extracts were tested using ELISA to reduce the expression of MMP-2 (Figure 3A,B) and MMP-9 proteins (Figure 3C,D). The results showed that both extracts significantly suppressed the synthesis of metalloproteinases in both of the tested cell lines. The ACE, at a higher concentration of 300 µg/mL for SW-480 cells (Figure 3A) and 600 µg/mL for HT-29 cells (Figure 3B), reduced the synthesis of MMP-2 by 28% and 50%, respectively, compared to the untreated control. The APE was even more effective. At the same concentrations, APE caused a 2-fold decrease in MMP-2 synthesis in HT-29 cells and a 4-fold decrease in SW-480 cells. Additionally, the inhibition was concentration-dependent and more effective than EGCG.

In SW-480 cells, the level of MMP-9 protein decreased by 40% after treatment with both ACE and APE at a concentration of 300 µg/mL (Figure 3C). In contrast, in HT-29 cells, MMP-9 secretion induced by 50 ng/mL TNF-α/TPA treatment was inhibited by 59% with a concentration of 600 µg/mL of ACE and 28% with APE at the same concentration (Figure 3D).

### 3.5. Effect of Aronia Leaf Extracts on MMP-2 and MMP-9 Activity—Gelatin Zymography

**Figure 4:**
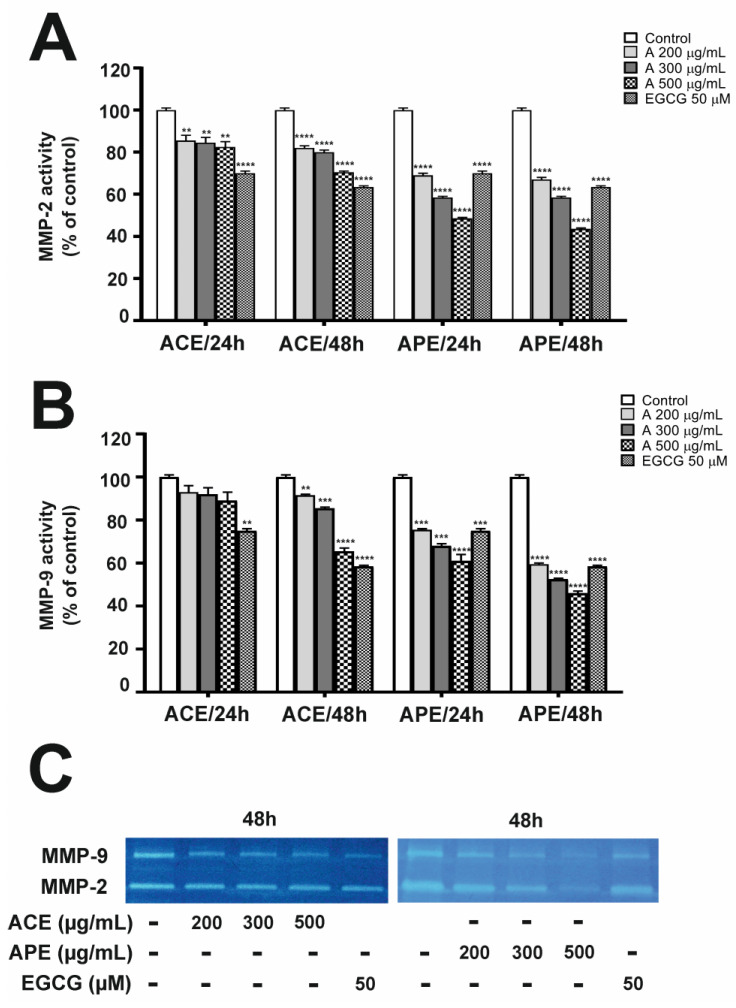
Aronia leaf extracts modulated MMP-2 (**A**) and MMP-9 (**B**) activities in SW-480 cells. The ACE and APE inhibitory effect was determined using zymography assays. The activity levels of MMPs were analyzed in culture supernatants. SW-480 cells were treated with aronia leaf extracts at 200, 300, and 500 µg/mL concentrations for 24 and 48 h. Negative and positive controls were obtained from untreated cells (CTRL) and cells treated with 50 µM EGCG cultured in a medium containing 3% FBS. The lower panel (**C**) shows the representative zymograms obtained for SW-480 cells after ACE and APE treatment for 48 h. Each value represents mean ± SEM, n = 3 independent experiments (each performed in duplicate). Results are expressed as a percentage of MMP levels compared to CTRL, calculated after scanning densitometry and computerized analysis of gels. Significance of differences between means: ** *p* < 0.01, *** *p* < 0.001, **** *p* < 0.0001 versus CTRL (one-way ANOVA followed by Newman–Keuls post hoc test).

**Figure 5:**
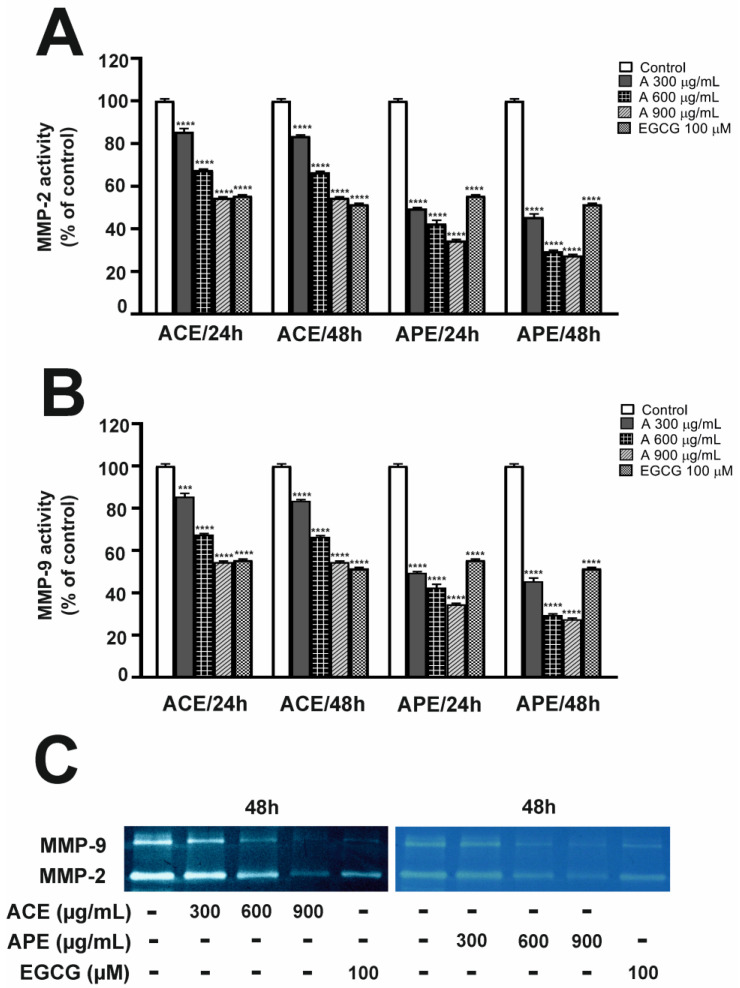
Aronia leaf extracts modulated MMP-2 (**A**) and MMP-9 (**B**) activities in HT-29 cells. The ACE and APE inhibitory effect was determined using zymography assays. The activity levels of MMPs were analyzed in culture supernatants. HT-29 cells were treated with aronia leaf extracts at 300, 600, and 900 µg/mL concentrations for 24 and 48 h. Negative and positive controls were obtained from cells stimulated with TNF/TPA (CTRL) and cells treated with 100 µM EGCG cultured in a medium containing 3% FBS. The lower panel (**C**) shows the representative zymograms obtained for HT-29 cells after ACE and APE treatment for 48 h. Each value represents mean ± SEM, n = 3 independent experiments (each performed in duplicate). Results are expressed as a percentage of MMP levels compared to CTRL, calculated after scanning densitometry and computerized analysis of gels. Significance of differences between means: *** *p* < 0.001, **** *p* < 0.0001 versus CTRL (one-way ANOVA followed by Newman–Keuls post hoc test).

As colorectal cancer progression involves the activity of MMP-2 and MMP-9, we investigated whether treatment with aronia extracts could affect gelatinase activity in SW-480 (Figure 4A,B) and HT-29 cells (Figure 5A,B). In the case of this analysis, we added higher concentrations (500 µg/mL for SW-480 cells and 900 µg/mL for HT-29 cells) to emphasize the inhibition of MMP levels based on the extract concentration used. As shown by zymography, the activities of MMP-2 and MMP-9 secreted in the culture medium by CRC cells were reduced in a concentration-dependent manner after both 24 and 48 h incubation with aronia leaf extracts. The exposure to ACE and APE at a concentration of 300 μg/mL resulted in a similar reduction in MMP-2 signals in SW-480 (Figure 4A) and HT-29 (Figure 5A) cells, regardless of incubation time. Moreover, the APE was more potent in inhibiting MMP-9 activity than the ACE at all concentrations tested and for both cell lines. The APE was also more effective in downregulating MMP-9 activity in HT-29 than in SW-480 cells (Figure 4B and Figure 5B). Interestingly, higher APE concentrations resulted in a more potent inhibition of gelatinase activities than EGCG.

## 4. Discussion

Data from the literature suggest that consuming dietary polyphenols regularly is a promising strategy for preventing and treating many types of cancer [39]. This is why specific polyphenols and natural plant extracts rich in these bioactive compounds continue to capture the interest of scientists worldwide. We focused on chokeberry leaf extracts, which have already shown significant antioxidant and cytotoxic properties against colon cancer cells [33].

Thanks to the obtained results, we were able to select the concentrations to be used in the current work. In the present study, we used the same aronia leaf extracts as the previous one: crude aronia leaf extract (ACE) and purified phenolic-rich aronia leaf extract (APE). Briefly, as we have demonstrated, chemical analysis of both extracts revealed the presence of several polyphenols, mainly polyphenolic acids and flavonols (Table 1). Chlorogenic acid (16.25 and 156.48 mg/g) and quercetin 3-rutinoside (1.99 and 39.79 mg/g) were the major compounds of both ACE and APE, respectively. The APE obtained from the ACE during the purification process resulted in a significant increase in the identified phenols: 42 in APE and 25 in ACE. The largest differences were detected between flavan-3-ols levels found in the APE at a concentration of 19.20 mg/g, while they were undetectable in ACE [33].

Data reported in this study found, for the first time, that both aronia leaf extracts effectively modulated the migration ability and the invasiveness of colon cancer cells (SW-480 and HT-29).

Furthermore, this effect could be attributed to a decrease in MMP-2 and MMP-9 gene expression at the protein level and their activity level following treatment with aronia extracts.

Research on cell migration and invasion in cancer is particularly interesting because metastatic progression is a significant cause of death in colon cancer patients. This is especially relevant because CRC often develops for a long time without symptoms, leading to a late diagnosis. Therefore, developing new therapeutic agents to prevent or tackle the spread of CRC is crucial. We conducted a Transwell assay to confirm that extracts from aronia leaves affect the migration and invasion of cancer cells. The results showed that both extracts significantly downregulated the motility of tested cells in a concentration-dependent manner. The migration of CRC cells was most efficiently decreased by an APE concentration of 100 µg/mL. Additionally, SW-480 cells showed greater sensitivity to both extracts compared to HT-29 cells. The results for cell invasion were similar to those of migration, with inhibition being higher in the SW-480 than in the HT-29 cells. This difference may partly be explained by the fact that the SW-480 cell line represents moderately differentiated cells with a high invasive capacity [40]. Our findings are consistent with previous studies in which aronia leaf extract reduced cell growth and was responsible for the concentration-dependent migration inhibition in human hepatoma cells (SK-Hep1) [41]. When SK-Hep1 cells were exposed to 200 μg/mL aronia leaf extract for 24 h, up to a 96.3% wound size inhibition was observed. Another in vitro study confirmed the reduction in the migratory and invasive capacities of colon cancer HCT116 cells after treatment with the leaf extract of *Thymus vulgaris* [42]. The effect of another leaf extract from *Annona muricata* on the motility and invasion potential in CRC cells was determined by Moghadamtousi et al. [43]. Research has confirmed that the tested extract markedly inhibited the migration and invasion of HT-29 and HCT116 cells. Similarly, treatment with *Terminalia catappa* leaf extract suppressed the invasion and migration of melanoma cells and inhibited MMP-2 activity in a concentration-dependent manner [44]. Moreover, Lotus leaf extract, which contains polyphenols such as quercetin, rutin, and kaempferol (also found in a significant amount in aronia leaves), significantly attenuated breast cancer cell migration [45]. The research findings discussed in this paper align with the results of other studies regarding the role of polyphenols and their extracts, not just those obtained from leaves. This highlights the significance of polyphenols as anti-metastatic agents in CRC. For example, 50 µM luteolin significantly reduced the migration and invasion of HT-29 and SW-480 cells. A simulation lasting 24 h notably downregulated the protein expression levels of MMP-2, MMP-3, MMP-9, and MMP-16, as detected by Western blotting [46]. The anticancer properties of silibinins investigated using CT26 mouse colon cell lines revealed a reduction in cell proliferation, survival, angiogenesis, and migration [47]. In another in vitro study, rosmarinic acid (RA) facilitated epithelial–mesenchymal transition (EMT) by upregulating the expression of the epithelial marker E-cadherin while reducing the expression of mesenchymal markers associated with aggressive tumor features, such as N-cadherin, Snail, Twist, or vimentin. Treatment with RA suppressed the invasion and migration of CRC cells (human HCT116 and mouse CT26) and decreased the expressions of MMP-2 and MMP-9 [48]. In contrast, Hung and coworkers reported no impact of commercial apple extract (65.7% procyanidins, 12.5% flavan3-ols, 6.5% other flavonoids, 10.8% nonflavonoids) at concentrations of 0.1–1.0 mg/mL on CRC DLD-1 cell viability. However, at concentrations of 500 µg/mL and 700 µg/mL, the extract inhibited the migration and invasion of DLD-1 cells and reduced their potential for colony formation and adhesion to a laboratory extracellular matrix model. These anti-metastatic properties were found to be a result of the inhibition of the regulator of growth factor receptor (FAK) signaling cascades and the disruption of the integrity of microtubules and actin fibers, which play an important role in cell motility and adhesion [49]. Brown et al. showed that berry extracts at physiologically relevant doses (0–50 µg/mL gallic acid equivalents) may influence the migration and invasion in HCT115 cells. The berry extracts significantly inhibited the invasive capabilities of CRC cells, even after simulating digestion and fermentation processes, but they did not reduce cell migration. The authors also suggested that the anti-invasive effect was unlikely to be related to cell motility but rather adhesion or enzymatic degradation [50]. Other research indicated that Liofenol™, a red wine extract composed of proanthocyanidins, flavonoids, and anthocyanins, resulted in more than a threefold increase in E-cadherin levels in HCT116 cells when applied at a concentration of 600 mg/mL. The loss of E-cadherin as a hallmark of EMT is associated with metastatic potential [51]. The study by Derry et al. investigated the characterization of azoxymethane (AOM)-induced lung metastasis of colon tumors in mice and evaluated the efficacy of grape seed extract (GSE) in preventing metastasis [52]. The mouse model used in the study was designed to replicate sporadic human CRC, which is crucial for understanding the metastasis process in the context of this tumor. GSE demonstrated the ability to inhibit metastasis, which was demonstrated by a reduction in the number of metastatic lung lesions and a slowing of tumor growth. This indicates that GSE may serve as an adjuvant in preventing metastasis of CRC.

We can also hypothesize that the observed effect of aronia leaf extracts tested in this study is possibly due to a complex interaction between the compounds in the extracts. It has been reported that the synergy of phytochemical mixtures produces a better response than individual compounds’ reduced expression of invasion-related molecules, including MMP-2 and MMP-9 [53]. It may also be the effect of the action of chlorogenic acid (CGA)—the main bioactive ingredient identified in tested chokeberry extracts, especially in the purified APE. Several scientific papers, including ours, show that plant extracts are a valuable source of CGA, a polyphenol with potential anticancer functions [54,55,56].

Our previous study showed that Japanese quince leaf phenol-rich extract (PRE), where CGA was also the dominant compound, significantly inhibited human colon cancer cell metastasis [55]. We demonstrated that PRE effectively reduced migration and invasion by suppressing the activity and protein expression of MMP-2 and MMP-9. Another extract rich in CGA isolated from *Lonicera japonica* Thunb., *Agrimonia eupatoria* L., and *Scutellaria barbata* D.Don positively attenuated the proliferation and migration capacity of the HCT116, HCT15, and CT26 colon cancer cell lines [57].

The anti-metastatic properties of chokeberry leaf extracts may also result from quercetin and its derivatives, the significant content of which was confirmed by HPLC analysis of the tested extracts. This flavonol is most abundant in food, especially leafy plants [58]. Numerous in vitro and in vivo studies have reported a robust concentration-dependent association between quercetin and decreased risk of colorectal cancer [59,60,61]. Han et al. indicated that quercetin (5 μM) remarkably suppressed the migratory and invasive capacity of colon cancer Caco-2 cells. Moreover, quercetin decreased the synthesis level of metastasis-related proteins MMP-2 and MMP-9 in a concentration-dependent manner [62]. In another study, quercetin in combination with *Lycopodium clavatum* extract resulted in a significant downregulation of MMP-2 and MMP-9 activities, as tested by gelatin zymography in colorectal cancer Colo-320 cells [63].

Additionally, research indicates that quercetin could be potentially used in the treatment of CRC with KRAS mutations [64]. KRAS mutations occur in approximately 40% of CRC cases and are associated with advanced disease status, poor tumor differentiation, distant metastasis, and resistance to chemotherapy [65]. Yang et al. found that KRAS-mutant cells (such as SW-480, DLD-1, or HCT116) were more sensitive to quercetin treatment than cells with wild-type KRAS (such as HT-29 or Colo-205). This effect was partly due to the induction of extrinsic and intrinsic apoptosis, possibly associated with the activation of JNK together with the inhibition of AKT signaling [64]. This may also explain why SW-480 cells showed greater sensitivity to the tested aronia leaf extracts compared to HT-29 cells.

Available studies reveal that MMP-2 and MMP-9 are involved in the aggressiveness of colon cancer [66,67,68]. Therefore, we carried out enzyme-linked immunosorbent assays (ELISAs), which confirmed the downregulation of MMP-2 and MMP-9 protein synthesis following exposure to aronia leaf extracts. However, to determine the changes in the MMP-9 protein level in HT-29 cells, we had to stimulate the cells with 50 ng/mL TNF-α/TPA. This resulted in a marked increase in MMP-9 expression, which was not observed under basal conditions. Our results agree with those previously reported by Lee et al., who investigated the impact of *Diospyros kaki* leaf extract (PLEO) on the activity and expression levels of MMP-2 and MMP-9 in colon cancer cells [69]. Additionally, the authors revealed that PLEO extract suppressed the migratory and invasion properties of colon cancer HT-29 and HCT116 cells.

Next, we conducted gelatin zymography analysis to further study changes in the levels of MMP-2/-9 secretion. This method provides a good alternative for estimating gelatinase activity in CRC cells compared to Western blot or ELISAs. We found that both extracts conspicuously suppressed the activity of metalloproteinases in both CRC cells. This suggests that the decrease in MMP-2 and MMP-9 activities can be attributed to suppressing their protein synthesis level, mediated by secreted bioactive compounds from aronia leaf extracts. The effective inhibition of MMP-2 and MMP-9 activity and cell migration was also verified in DU145 prostate cancer cells using a *Psidium guajava* leaf extract rich in rutin and quercetin, which were also the dominant components of our tested extracts [70]. Additionally, *Hibiscus sabdariffa* leaf extract (HLE) effectively inhibited the activity and protein expression level of MMP-9 in human prostate cancer LNCaP cells in a concentration-dependent manner [71]. However, the HLE did not have any influence on MMP-2 expression. HPLC analysis revealed that the HLE contained, among others, procyanidin B2, rutin, and quercetin. In conclusion, not all leaf extracts can decrease the activity of both MMP-2 and MMP-9 in cancer, which emphasizes the value of the tested chokeberry leaf extracts. The preventive and therapeutic effectiveness of polyphenols found in aronia leaf extracts as modulators of MMPs in CRC still requires further investigation due to certain limitations in the existing research. Cancer cell lines are widely used to study various aspects of CRC biology. Although the results indicate that natural polyphenols and their extracts may potentially prevent CRC, the concentrations established in these preclinical studies are often not reflected in vivo and clinical studies. The discrepancy between results may be because cancer cell lines are generally cultured in an artificial environment, which does not fully reflect the three-dimensional, often hypoxic environment in which tumor development takes place. On the other hand, this allows for the strict control of conditions, ensures the repeatability of results, and permits the testing of specific concentrations, but it cannot accurately reproduce human conditions. Even though the concentrations of polyphenols obtained from in vitro studies are often higher than those obtained under natural conditions in the intestine, these studies provide a foundation for further research and hold significant cognitive value. Another limitation is the heterogeneity of numerous factors, such as environmental influences, geographic location, the timing of harvest or storage conditions, or even the method of extraction and solvent used, which may affect the polyphenol content in aronia leaves and their extracts. Consequently, new formulations or delivery systems that enhance their bioavailability and bioaccessibility are needed. Nonetheless, considering their properties and metabolism, polyphenols hold promise primarily as chemopreventive agents rather than therapeutic ones. New research involving many animal models and clinical trials is essential to show the exact mechanisms of polyphenols and validate their efficacy as inhibitors of MMP-2 and MMP-9 in CRC.

## 5. Conclusions

The findings indicate a pharmacological rationale for further exploring the biological potential of aronia leaf extracts that may regulate and impact certain aspects of colon cancer cell metastasis. This study was the first to investigate the influence of these extracts on the expression of matrix metalloproteinases MMP-2 and MMP-9. The results demonstrated that APE proved to be more effective than ACE in inhibiting the migration and invasiveness of the tested CRC cells. This effect was particularly notable in SW-480 cells. Furthermore, both extracts significantly inhibited MMP-2 and MMP-9 protein synthesis levels in SW-480 and HT-29 cells. The activities of gelatinases were similar for both CRC lines, and the APE, tested at a concentration of 300 µg/mL, nearly reached the IC50 value after 48 h of incubation. A question of the possible applications of aronia leaf extracts in preventing the occurrence and development of CRC remains open to validation by animal models.

## Figures and Tables

**Table 1:** Composition of *Aronia melanocarpa* leaf extracts (ACE and APE) [33].

Group of Compounds [mg/g]	Identity	ACE	APE
Phenolic acids Total	Caffeoylquinic acid 3-*O*-caffeoylqunic acid Dicaffeoylquinic acid I *p*-coumaroylquinic acid I Dicaffeoylquinic acid II Chlorogenic acid 4-*O*-caffeoylqunic acid *p*-coumaroylquinic acid II	- 5.78 ± 0.16 - 0.60 ± 0.03 - 16.25 ± 0.94 0.64 ± 0.03 0.34 ± 0.01 23.61 ± 1.17	0.94 ± 0.00 31.05 ± 0.07 0.36 ± 0.00 7.88 ± 0.02 0.42 ± 0.00 156.48 ± 0.12 7.30 ± 0.02 - 204.43 ± 0.23
Flavonols Total	Quercetin 3-rutinoside 7-glucoside Quercetin dihexoside I Quercetin dihexoside II Quercetin dirhamnosylhexoside I Quercetin dirhamnosylhexoside II Quercetin 3-vacianoside I Quercetin rhamnosylhexoside Quercetin 3-vacianoside II Quercetin 3-robinobioside Quercetin 3-rutinoside Quercetin 3-glucoside Quercetin 3-galactoside Quercetin coumaroylglucoside Kaempferol coumaroylglucoside Isorhamnetin 3-rutinoside Isorhamnetin rhamnosylhexoside isomer	- 0.61 ± 0.03 0.11 ± 0.00 0.07 ± 0.00 - - - 0.89 ± 0.04 0.40 ± 0.02 1.99 ± 0.08 1.15 ± 0.01 0.48 ± 0.02 0.07 ± 0.00 0.10 ± 0.01 0.12 ± 0.00 0.15 ± 0.01 6.14 ± 0.22	0.48 ± 0.00 12.61 ± 0.01 3.67 ± 0.00 2.62 ± 0.00 2.16 ± 0.00 0.69 ± 0.01 0.53 ± 0.01 17.68 ± 0.02 8.06 ± 0.01 39.79 ± 0.17 21.38 ± 0.23 9.74 ± 0.01 2.17 ± 0.04 1.77 ± 0.00 2.54 ± 0.00 3.32 ± 0.00 129.21 ± 0.51
Flavanols Total	Procyanidin B2 Cyanidin 3-glucoside (-)-Epicatechin	- 0.08 ± 0.01 - 0.08 ± 0.01	16.56 ± 0.02 7.86 ± 0.19 2.56 ± 0.01 26.98 ± 0.22

## Data Availability

The raw data supporting the conclusions of this article will be made available by the authors upon request.
